# Quality of Evidence Supporting the Role of Curcuma Longa Extract/Curcumin for the Treatment of Osteoarthritis: An Overview of Systematic Reviews

**DOI:** 10.1155/2022/6159874

**Published:** 2022-03-31

**Authors:** Wenqiang Chen, Hongshuo Shi, Pin Deng, Zhenguo Yang, Wenbin Liu, Lu Qi, Chengda Dong, Guomin Si, Dong Guo, Lei Wang

**Affiliations:** ^1^College of Traditional Chinese Medicine, Shandong University of Traditional Chinese Medicine, Jinan, China; ^2^The Third Affiliated Hospital of Beijing University of Chinese Medicine, Beijing, China; ^3^The Second Affiliated Hospital of Shandong University of Traditional Chinese Medicine, Jinan, China; ^4^First Clinical Medical College, Shandong University of Traditional Chinese Medicine, Jinan, China; ^5^Department of Traditional Chinese Medicine, Provincial Hospital Affiliated to Shandong First Medical University, Jinan, China; ^6^Center for Faculty Development, Shandong University of Traditional Chinese Medicine, Jinan, China

## Abstract

**Background:**

Well known for its good anti-inflammatory effect, curcuma longa extract (CLE)/curcumin (C) has a potential effect on osteoarthritis (OA), and a large number of researchers have completed several systematic reviews/meta-analyses (SRs/MAs) in this research area. However, the methodological and evidentiary quality of these SRs/MAs need to be further evaluated, and whether these findings provide reliable evidence for clinicians remains controversial.

**Methods:**

Two researchers collected data from seven databases for SRs/MAs that are about randomized controlled trials (RCTs) on CLE/C for OA. Assessment was made for the SRs/MAs included in this article by means of the Assessment System for Evaluating Methodological Quality 2 (AMSTAR-2), the Risk of Bias in Systematic (ROBIS) scale, the list of Preferred Reporting Items for Systematic Reviews and Meta-Analyses (PRISMA), and the Grading of Recommendations Assessment, Development, and Evaluation (GRADE) system.

**Results:**

Nine published SRs/MAs were included in our study. According to the results of the AMSTAR-2 assessment, only one SR/MA was assessed as high quality. According to the ROBIS evaluation results, only 2 SRs/MAs have a low risk of bias. According to the results of the PRISMA checklist assessment, only 2 SRs/MAs studies fully reported the checklist, while other studies had reporting flaws. According to GRADE, a total of 59 effect sizes extracted from the included SRs/MAs were evaluated, among which no effect size was rated as high.

**Conclusions:**

CLE/C may be an effective and safe complementary treatment for OA. However, further standard SRs/MAs and RCTs are needed to provide an evidence-based medical rationale for this.

## 1. Introduction

Osteoarthritis (OA) is a global inflammatory joint disease. It is one of the main causes of joint disability [1]. With the combined effects of aging, rising obesity rate, and the increase in the number of joint injuries, OA has become an increasingly common disease on a worldwide scale, and a global estimate indicates that the affected population has reached 250 million. [2, 3]. Pain, joint stiffness or deformity, and even terminal disability are commonly reported as typical symptoms of OA in studies around the world [4, 5]. However, most current drug therapies focus only on pain relief and symptomatic treatment, including the use of nonsteroidal anti-inflammatory drugs (NSAIDs), intra-articular injection of glucocorticoids and opioids [6]. However, gastrointestinal discomfort and dose dependence are common problems with these drugs [7, 8]. In addition, total knee arthroplasty (TKA) is mainly used to treat severe KOA, which has a high complication rate [9]. Therefore, it is necessary to explore a safer treatment for OA.

OA used to be considered a degenerative cartilage disease, but now, this concept has been transformed into a complex disease that affects the entire joint [10]. It is now recognized that OA involves mechanical, inflammatory, and metabolic factors, rather than a simple “wear and tear” disease. Inflammation plays a greater role in the pathogenesis of OA than previously recognized, and OA is now regarded as a low-grade inflammatory disease that affects all tissues of the joint, including cartilage degeneration, bone remodeling, osteophytes, and synovitis [11]. In view of the fact that inflammation may play a key role in the pathogenesis and progression of osteoarthritis, it may be a good idea to develop new treatments for OA from this perspective.

Recently, curcuma longa extract (CLE, an anti-inflammatory and antioxidant preparation) has been used in traditional Chinese medicine and Ayurveda to treat arthritis and has thus become an attractive treatment option for improving the joint condition of OA patients [12]. Often used as an alternative medicine or dietary supplement, turmeric is typically an extract that is standardized to 80–95% curcuminoids, which include curcumin (C), demethoxylated curcumin (DMC), and didemethoxylated curcumin (BDMC), among which C [13] is the most active ingredient in turmeric and is “generally regarded as safe” by the US FDA [14]. In addition, the CLE alone has anti-inflammatory properties similar to NSAIDs [15]. It has been shown that CLE affects the signal transduction of proinflammatory cytokines by influencing the activity of NF-*κ*B, such as interleukin, phospholipase A2, 5-lipoxygenase, and COX-2 [16].

Many systematic reviews/meta-analyses (SRs/MAs) have been conducted to evaluate the potential therapeutic benefits of CLE/C for patients with OA. However, the conclusions are inconsistent due to the defects of the quality and methods of the preliminary research studies. The systematic review is a novel tool for solving specific and key issues related to policies and practices [17]. The purpose is to combine the evidence from multiple SRs/MAs to form a practical document that can be used to guide healthcare professionals and decision-makers [18]. The purpose of our research is to use a systematic overview to critically evaluate the scientific quality of related SRs/MAs in the CLE/C treatment of OA.

## 2. Materials and Methods

### 2.1. Protocol Registration

This overview protocol has been registered with the INPLASY website (Registration number: INPLASY202220063).

### 2.2. Research Methods

The SR/MA overview is based on the guidelines specified in Cochrane Handbook [19], the Preferred Reporting Project for System Reviews and Meta-Analyses (PRISMA) statement (Supplementary file 1) [20], and the overview of high-quality methods [21, 22].

### 2.3. Development of Inclusion and Exclusion Criteria

#### 2.3.1. Literature Inclusion Criteria

- Study Design: This overview only includes SRs/MAs from randomized controlled trials (RCTs) of CLE/C in the treatment of OA.
- Study Participants: Subjects who have been clinically or radiologically diagnosed with OA according to national or international standards, regardless of gender, race, or age.
- Study Intervention: The intervention method was CLE/C; the control group was treated with conventional treatment (CT) or placebo.
- Study Outcome Measures: Western Ontario and McMaster University Arthritis Index Score (WOMAC), visual analog scale (VAS), adverse reactions, and other outcome measures, including the use of rescue drugs, incidence of withdrawal from treatment due to adverse events, the use of rescue drugs, walking distance, and analgesic discontinuation rate.

#### 2.3.2. Exclusion Criteria

Duplicate publications, other overviews, conference abstracts, narrative reviews, and network meta-analysis were excluded.

### 2.4. Search Strategy

Two researchers (WQ-C and HS-S) independently conducted a literature search. The search was carried out with 7 databases including PubMed, Embase, Cochrane Library, CNKI, Wanfang Database, Chongqing VIP, and Chinese Biological Medicine (CBM) Database from its establishment until December 1, 2021. The search strategy adopts a combination of MeSH terms and free words. We searched the above databases with the following key terms: curcuma longa extract, curcumin, osteoarthritis, systematic reviews, and meta-analysis. We also manually searched the references of related articles. The specific search strategy was modified according to different databases. Supplementary file 2 provided the search strategy.

### 2.5. Eligibility Assessment and Data Extraction

Two researchers (WQ-C and P-D) independently performed literature screening. After deleting duplicate content, researchers read the title and abstract to find potential SRs/MAs based on the inclusion and exclusion criteria. Then, full-text articles were obtained for further screening to determine their eligibility. Afterwards, two researchers (ZG-Y and WB-L) independently extracted data using a standardized data extraction form. The following specific characteristics are extracted from each SR/MA: first author, the year of publication, country, the number of included studies, sample size, treatment intervention, control intervention, quality assessment methods, results, and main conclusions.

### 2.6. SRs/MAs Quality Assessment

Quality assessment of included SRs/MAs was performed independently by two researchers (Q-L and CD-D).

#### 2.6.1. Assessment of Methodological Quality

The Assessment System for Evaluating Methodological Quality 2 (AMSTAR-2) [23] scale was used to assess the methodological quality of the included SRs/MAs. It consists of 16 items, 7 of which are critical areas (2, 4, 7, 9, 11, 13, and 15). Each item was assessed and rated as “yes,” “partially yes,” or “no.”

#### 2.6.2. Assessment of Risk of Bias

The risk of bias of the included SRs/MAs was assessed by the risk of bias in systematic (ROBIS) scale [24]. The scale was completed in 3 stages to assess the overall risk of bias. The results are assessed and rated as “low,” “unclear,” or “high.”

#### 2.6.3. Assessment of Reporting Quality

The list of PRISMA was used to assess the quality of each SR/MA report based on the following aspects: (a) title, (b) summary, (c) introduction, (d) method, (e) result, (f) discussion, and (g) funding. It consists of 27 items, with a focus on reporting methods and results in a meta-analysis. Based on the completeness of the project information report, each project is assessed and rated as “yes” (full report), “partial yes” (partial report), or “no” (no report).

#### 2.6.4. Assessment of Quality of Evidence

The GRADE scale was used to assess the quality of the evidence of the included SRs/MAs from five aspects: research limitations, inconsistencies, indirectness, imprecision, and publication bias [25].

### 2.7. Data Synthesis and Presentation

In this overview, a narrative synthesis was used. The characteristics and results of each SR/MA and the assessment results of AMSTAR 2, PRISMA, ROBIS, and GRADE were reported in the form of a list.

## 3. Results

### 3.1. Results on Literature Search and Screening

**Figure 1:**
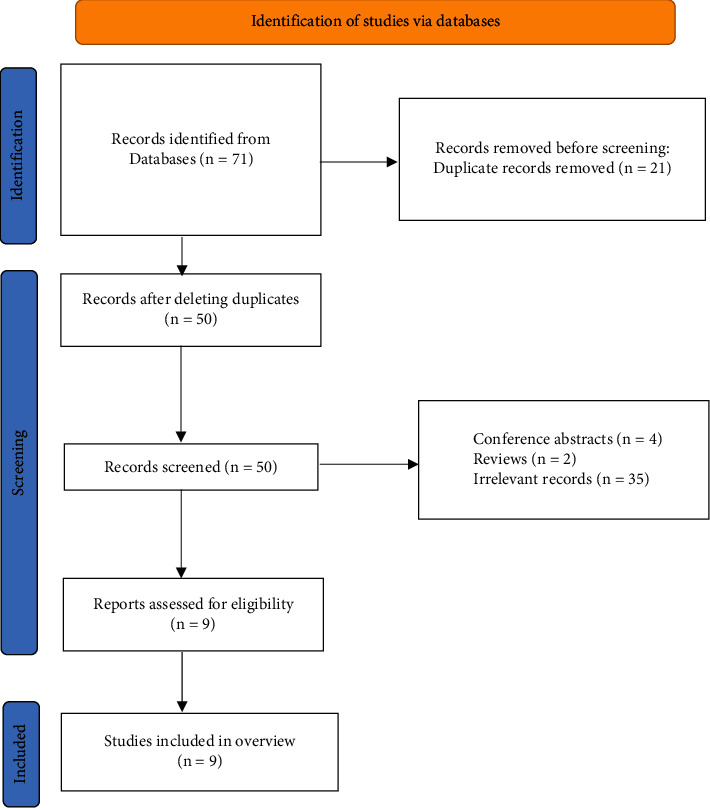
The flowchart of the screening process.

A total of 71 articles were retrieved from seven literature databases, and 21 duplicate articles were deleted. We filtered by the title and abstract of the literature and finally obtained 9 literature studies for full-text screening. After evaluation according to the inclusion and exclusion criteria, we finally obtained 9 literature studies [26, 27] included for study.  Figure 1 shows the screening flow chart.

### 3.2. Description of Included SRs/MAs

Nine SRs/MAs [26, 27] published from 2016 to 2021 were included, and all the included papers were SR and MA. Of these published SRs/MAs, five were from China [27–31], and the remaining four were from the United States [26], the United Kingdom [32], Australia [33], and South Korea, respectively [34]. Among them, 8 SRs/MAs [26, 28–34] were published in English and one [27] was published in Chinese. All SRs/MAs contained a total of 28 RCTs, and the number of RCTs included in each SR/MA ranged from 5 to 15, and the total number of individuals in the included RCTs for a single SR/MA ranged from 599 to 1,621. The intervention rendered to the treatment group was curcuma longa extract or curcumin, and the control group was treated with CT or placebo, and CT modalities include painkillers and NSAIDs. In terms of quality assessment scales, all the literature adopted the Cochrane risk of bias standard. The details of the SRs/MAs included are shown in Table 1.

### 3.3. Summary of the Results of the Included Studies

The result indicators extracted from the included studies are listed in Table 2.

#### 3.3.1. Efficacy and Safety of CLE/C for OA (Compared with Placebo Group) (Table 2(a))

*(1) Pain*. All SRs/MAs [26–34] have reported that CLE/C could significantly alleviate the pain of OA patients compared with placebo. Among them, 2 SRs/MAs [26, 33] gave a direct quantitative report that CLE/C could significantly relieve pain. Seven SRs/MAs [27–32, 34] reported that CLE/C could significantly reduce the VAS score of OA patients. Two SRs/MAs [29, 31] reported that CLE/C could significantly reduce the pain score of the WOMAC scale in patients with OA.

*(2) Function*. Nine SRs/MAs [26–34] reported that CLE/C could significantly improve the joint function of patients with OA. Among them, 2 SRs/MAs [26, 33] gave a direct quantitative report that CLE/C could significantly improve the joint function of patients with OA, and 5 of the SRs/MAs [27, 29, 30, 32, 34] reported that CLE/C could significantly improve the WOMAC scale for patients with OA. Two SRs/MAs both reported that CLE/C could significantly reduce the physical score [29, 31] and stiffness score [29, 31] of the WOMAC scale in patients with OA compared with placebo. In addition, 2 SRs/MAs reported that CLE/C could significantly improve the walking distance [27] and Lequesne pain-function index (LPFI) [32] of patients with OA, respectively.

*(3) Adverse events*. Seven SRs/MAs [26–31, 33] reported the occurrence of adverse events in CLE/C compared with placebo, none of which was statistically significant. Two SRs/MAs [26, 33] reported that, compared with placebo, there was no significant difference in the use of rescue drugs during the treatment with CLE/C. A SR/MA [26] reported that CLE/C was not statistically significant in the incidence of withdrawal from treatment due to adverse events compared with placebo.

#### 3.3.2. Efficacy and Safety of CLE/C for OA (Compared with CT) **(**Table 2(b))

*(1) Pain*. Five SRs/MAs [26–28, 31, 33] reported the effects of CLE/C on pain in patients with OA compared with NSAIDs, and 2 SRs/MAs [31, 33] indicated that CLE/C had similar effects on pain relief compared with NSAIDs. In addition, a SR/MA [27] showed that NSAIDs were superior to CLE/C in reducing pain in patients with OA.

*(2) Function*. Seven SRs/MAs [26–28, 30–34] reported the improvement of joint function between CLE/C and CT in patients with OA. One SR/MA [34] reported that CLE/C exhibited no statistical significance in the improvement of joint function compared with analgesics. The results of the other 6 SRs/MAs [26–28, 30–33] also showed that CLE/C was equivalent to NSAIDs in improving joint function. One SR/MA [27] reported that there was no difference between CLE/C and NSAIDs in improving walking distance.

*(3) Adverse events*. Six SRs/MAs [26–28, 30–33] reported the occurrence of adverse events in CLE/C compared with NSAIDs. Among them, the results of 4 SRs/MAs [26, 27, 31, 33] showed that patients using CLE/C had a lower incidence of adverse events. Two SRs/MAs [26, 33] reported no difference between CLE/C and NSAIDs in the use of rescue drugs. A SR/MA [26] reported that CLE/C has a lower incidence of withdrawal due to adverse events than NSAIDs.

### 3.4. Results on SRs/MAs Quality Assessment

#### 3.4.1. Methodological Quality Assessment

The AMSTAR-2 assessment breakdown for each review is shown in Table 3. Only one SR/MA was of high quality [29]. Since more than one key item was missing in the remaining SRs/MAs, their quality was rated very low. The method restriction came from the following items: item 2 (only 2 SRs/MAs [26, 29] had registered in the protocol), item 7 (only 2 SRs/MAs provided a research exclusion list), and item 15 (the 4 SRs/MAs [26, 27, 30, 32] did not conduct publication bias studies or discuss their impact on SR/MA).

#### 3.4.2. Risk of Bias of the Included SRs/MAs

The risk of bias for all SRs/MAs [26, 29] in the first stage and Domain 1 of the ROBIS evaluation was assessed as low risk. In Domain 2, 6 SRs/MAs [26, 27, 29, 30, 32, 33] were assessed as low risk. In Domain 3, 8 SRs/MAs [26, 28–34] were assessed as low risk of bias and only 2 SRs/MAs [28, 29] were assessed as low risk of bias in Domain 4. In Phase 3, 7 SRs/MAs [26, 28–31, 33, 34] had a low risk of bias. The evaluation details of the included SRs/MAs on the ROBIS scale are shown in Table 4.

#### 3.4.3. Report Quality

The results of the PRISMA inventory evaluation are shown in Table 5. Among the 27 items, 24 items had a “yes” response rate of more than 70%, which showed that the report was relatively complete. However, there were some reporting deficiencies in other projects. Items 5 (protocol and registration) was inadequately reported (the “yes” response rate is less than 50%).

#### 3.4.4. Evidence Quality

Table 6 shows the results of GRADE evaluation including SR/MA-related effect sizes. The 9 SRs/MAs included 59 effect sizes related to the efficacy and safety of CLE/C for OA. In the evaluation results based on the effect sizes, 19 were rated as medium, 20 low, and 20 very low in terms of the quality of evidence. Publication bias (*n* = 42) was the most common downgrading factor, followed by imprecision (*n* = 38), inconsistency (*n* = 23), risk of bias (*n* = 22), and imprecision (*n* = 0).

## 4. Discussion

CLE/C may be a complementary treatment for OA, which is a common disease in the elderly. At the same time, more and more related SRs/MAs have been carried out. This overview summarizes the available evidence to comprehensively assess the efficacy of CLE/C for the treatment of OA.

### 4.1. Summary of the Main Findings

This overview incorporated 9 SR/MAs published between 2016 and 2021, 8(8/9, 88.9%) of which were published in the past 5 years, indicating that an increasing attention has been paid to the effectiveness and safety of CLE/C treatment of OA in recent years.

Based on the results of the AMSTAR-2 evaluation in this overview, only one methodological quality of SR/MA was evaluated as high, and the other methodological qualities were very low, and especially in items 2 (protocol registration, 2/9, 22.2%), 7 (exclusion list, 1/9, 11.1%), and 15 (publication bias, 55.6%). Only 2 SRs/MAs were registered for the protocol. Protocol registration is very important when conducting each SR/MA and should be carried out at the time of topic selection, which helps reduce the potential for selective reporting bias and ensures that each SR/MA is conducted in a more accurate manner [35]. Only 2 SRs/MAs provided a complete list of excluded literature, increasing publication bias. Providing a list of excluded literature can provide strong evidence of the accuracy and rigor of the literature screening process. In addition, publication of biased assessments may reduce the veracity of the final results. In this overview, we used the ROBIS scale to evaluate the risk of bias for the included SRs/MAs. Among them, incomplete literature retrieval and insufficient evaluation of publication bias are the main factors leading to a high risk of bias. Similar to the results of the AMSTAR-2 evaluation, the evaluation using the PRISMA checklist found that only 2 SRs/MAs completed the program registration.

The quality of the evidence is based on the GRADE system. Among the 59 effect sizes, no one was rated as high in terms of the quality of evidence. Publication bias is the most common downgrading factor, followed by imprecision, inconsistency, risk of bias, and imprecision. Further analysis revealed a lack of publication bias analysis or the presence of publication bias for the effect sizes included in the SRs/MAs included in this overview. In addition, the insufficient number of RCTs included in the publication bias assessment of the relevant effect size is a potential reason for the missing publication bias assessment. When assessing the quality of evidence for a relevant effect size, the insufficient number of study populations included in that effect size is also a significant contributor to the low quality of the final evidence. Descriptive analysis shows that CLE/C is an effective treatment for OA and may be safer than CT. Due to the low methodological and evidentiary quality of the included RCTs, conclusions from the inclusion of SRs/MAs may differ from real-world outcomes and caution should be exercised when recommending CLE/C as a complementary intervention for OA.

### 4.2. Implications for Future Research

Various aspects of each SR/MA included were assessed using AMSTAR-2, PRISMA, ROBIS, and GRADE with the aim of facilitating future standardization of SRs/MAs. Researchers conducting SRs/MAs should register or publish study protocols in advance to minimize the risk of bias and ensure the credibility of SRs/MAs results and should provide a list of excluded literature with explanations to ensure transparency and minimize publication bias. In future RCTs, it is important to increase the sample size of the study in a reasonable way to increase the credibility of the evidence. In addition, a complete evaluation of publication bias will also increase the accuracy of the meta-analysis results. With the development of evidence-based complementary and alternative medicine, it is hoped that researchers will continue to promote the standardization of related single RCTs in the future. Well-designed and strictly implemented RCTs can minimize or avoid bias. This is the gold standard for evaluating interventions [36].

### 4.3. Strengths and Limitations

As far as we know, our study is the first overview of SRs/MAs on the use of CLE/C in the treatment of OA, which can provide a comprehensive evidence reference for clinical practice. In addition, the evaluation process of AMSTAR-2, PRISMA, ROBIS, and GRADE revealed the obvious limitations of SRs/MAs and RCT, which may help guide high-quality research in the future. However, we found that the methodological quality of most of the included SRs/MAs was poor, a limitation that also prevented our study from drawing firm conclusions about the use of CLE/C for OA.

## 5. Conclusion

According to the available published evidence, CLE/C may be effective and safe for the treatment of OA. However, due to the generally low quality of methodologies, reports, and evidence in the included SRs/MAs, clinicians should approach this finding with caution in their practice.

## Figures and Tables

**Table 1:** Characteristics of the included SRs/MAs.

Author, year (country)	Trials (subjects)	Intervention group	Control group	Quality assessment	Main results
Raveendhara R. 2018 (USA) [26]	7 (769)	Curcumin	CT, placebo	Cochrane	Curcumin compounds can be a valuable supplement to the pharmacological treatment of OA in relieving pain, improving physical function, and reducing the risk of adverse events.
James W. Daily 2016 (South Korea) [34]	8 (892)	Curcumin, curcuma longa extract	CT, placebo	Cochrane	Compared with placebo, turmeric/curcumin can significantly reduce the VAS and WOMAC scores of OA patients. Compared with analgesics, the VAS scores of turmeric/curcumin and the control group were not significantly different, and there was no significant difference in the occurrence of adverse events.
An-Fang Hsiao 2021 (China) [28]	11 (1,258)	Curcumin	CT, placebo	Cochrane	Curcumin compounds were significantly better than the control drugs in the VAS score and WOMAC pain score, and there was no significant difference in the occurrence of adverse events.
Igho J. ONAKPOYA 2017 (UK) [32]	7 (797)	Curcumin	CT, placebo	Cochrane	Compared with placebo, curcuma longa extract has obvious effect in relieving pain and improving physical function. However, compared with NSAIDs, curcuma longa extract cannot improve stiffness and has less pain relief effect for patients with knee OA.
Zhiqiang Wang 2021 (Australia) [33]	10 (1,810)	Curcuma longa extract	CT, placebo	Cochrane	Compared with placebo, curcuma longa extract has obvious effect in relieving pain and improving physical function. However, compared with NSAIDs, curcuma longa extract has a higher level of safety for patients with knee OA.
Wenli Dai 2021 (China) [29]	10 (783)	Curcuma longa extract	Placebo	Cochrane	Compared with placebo, curcuma longa extract is more beneficial in relieving pain and improving the symptomatic OA, and there is no difference in the risk of adverse reactions.
Jian Wu 2019 (China) [30]	5 (599)	Curcumin, curcuma longa extract	CT, placebo	Cochrane	Curcumin can effectively treat patients with OA, improve WOMAC score and VAS score, and curcumin has no more side effects than ibuprofen.
Liuting Zeng 2021 (China) [31]	15 (1,621)	Curcumin, curcuma longa extract	CT, placebo	Cochrane	Both curcuma longa extract and curcumin can relieve pain and joint stiffness in patients with OA, improve joint function, and will not increase the occurrence of adverse events.
Weiyan Gong 2017 (China) [27]	6 (606)	Curcumin, curcuma longa extract	CT, placebo	Cochrane	Curcumin has the effect of treating OA without increasing gastrointestinal side effects

**Table 2:** Summary of evidence.

Author, year (country)	Outcomes	Studies (participants)	Relative effect (95% CI)	Heterogeneity
*(a) (CLE/C vs placebo)*				
Raveendhara R. 2018 (USA) [26]	Pain	5 (331)	SMD: −0.81 (−1.25, −0.37)^∗^	*I*2 = 71%
	Function	3 (232)	SMD: −0.48 (−0.74, −0.22)^∗^	*I*2 = 0%
	The use of rescue drugs	3 (141)	RR: 0.65(0.48, 1.05)	*I*2 = 74%
	Incidence of withdrawal from treatment due to adverse events	4 (288)	RR: 0.90 (0.21, 3.79)	*I*2 = 14%
	Adverse events	3 (247)	RR: 2.22 (0.94, 5.26)	*I*2 = 0%
James W. Daily 2016 (South Korea) [34]	VAS	3 (104)	MD: −2.04 (−2.85, −1.24)^∗^	*I*2 = 27%
	WOMAC scale	3 (122)	MD: −15.36 (−26.9, −3.77)^∗^	*I*2 = 91%
An-Fang Hsiao 2021 (China) [28]	VAS	7 (501)	SMD: −2.073 (−4.339, 0.194)	*I*2 = 96.6%
	Adverse events	6 (527)	Or: 1.115 (0.548, 2.271)	*I*2 = 0%
Igho J. ONAKPOYA 2017 (UK) [32]	VAS	5 (366)	SMD: −3.30(−4.99,−2.01)^∗^	*I*2 = 97%
	WOMAC scale	3 (167)	SMD: −4.42 (−6.66, −2.19)^∗^	*I*2 = 93%
	LPFI	2 (107)	MD: −2.69 (−3.48,−1.90)^∗^	*I*2 = 0%
Zhiqiang Wang 2021 (Australia) [33]	Pain	12 (1,071)	SMD = −0.82 (−1.17, −0.47)^∗^	*I*2 = 86.23%
	Function	10 (973)	SMD = −0.75 (−1.18, −0.33)^∗^	*I*2 = 90.05%
	Adverse events	8 (791)	RD: 0.00 (−0.06,0.06)	*I*2 = 31.85%
	The use of rescue drugs	7 (300)	RD: −0.13 (−0.24,−0.01)^∗^	*I*2 = 54.36%
	Analgesic discontinuation rate	4 (154)	RD: 0.36 (0.1, 0.61)^∗^	*I*2 = 87.06%
Wenli Dai 2021 (China) [29]	VAS	8 (569)	MD: −2.21 (−3.15, −1.28)^∗^	*I*2 = 94%
	WOMAC scale	5 (377)	MD: −11.93 (−16.63, −7.23)^∗^	*I*2 = 81%
	WOMAC (pain) scale	5 (377)	MD: −1.94 (−2.80, −1.09)^∗^	*I*2 = 76%
	WOMAC (physical) scale	5 (377)	MD: −6.45 (−9.10,−3.80)^∗^	*I*2 = 83%
	WOMAC (stiffness) scale	5 (377)	MD: −0.53 (−0.95, −0.11)^∗^	*I*2 = 77%
	Adverse events	7 (623)	RR: 1.08 (0.69, 1.70)	*I*2 = 19%
Jian Wu 2019 (China) [30]	WOMAC scale	3 (146)	SMD: −1.30 (−1.66, −0.94)^∗^	*I*2 = 37%
	VAS	2 (98)	SMD: −1.65 (−2.11, −1.19)^∗^	*I*2 = 0%
	Adverse events	2 (113)	RR:1.46 (0.57, 3.77)	*I*2 = 0%
Liuting Zeng 2021 (China) [31]	VAS	6 (381)	MD: −11.55 (−14.3, −9.06)^∗^	*I*2 = 69%
	WOMAC (pain) scale	4 (315)	SMD: −0.66 (−0.88, −0.43)^∗^	*I*2 = 34%
	WOMAC (physical) scale	4 (315)	SMD: −0.79 (−1.27, −0.31)^∗^	*I*2 = 75%
	WOMAC (stiffness) scale	4 (315)	SMD: −0.35 (−0.57, −0.12)^∗^	*I*2 = 26%
	Adverse events	6 (629)	RR: 1.18 (0.71, 1.94)	*I*2 = 25%
Weiyan Gong 2017 (China) [27]	VAS	2 (82)	SMD: −0.69 (−0.99, −0.40)^∗^	*I*2 = 48.4%
	WOMAC scale	2 (82)	SMD: −1.44 (−1.91, −0.96)^∗^	*I*2 = 0%
	Adverse events	2 (152)	Or: 1.5 (0.65, 3.44)	*I*2 = 0%
	Walking distance	1 (48)	MD: 202.0 (187.56, 216.44)^∗^	NA

*(b) (CLE/C vs CT)*				
Raveendhara R. 2018 (USA) [26]	Pain (vs NSAIDs)	2 (422)	SMD: −0.05 (−0.41, 0.31)^∗^	*I*2 = 60%
	Function (vs NSAIDs)	1 (331)	SMD: −0.02 (−0.24, 0.19)	NA
	The use of rescue drugs (vs NSAIDs)	2 (422)	RR 2.46 (0.48, 12.52)	*I*2 = 60%
	Incidence of withdrawal from treatment due to adverse events (vs NSAIDs)	2 (474)	RR: 0.22 (0.05, 0.99)^∗^	*I*2 = 0%
	Adverse events (vs NSAIDs)	2 (467)	RR: 0.74 (0.60, 0.91)^∗^	*I*2 = 0%
James W. Daily 2016 (South Korea) [34]	WOMAC scale (vs painkillers)	5 (625)	MD: −1.89 (−4.13,0.35)	*I*2 = 94%
An-Fang Hsiao 2021 (China) [28]	VAS (vs NSAIDs)	2 (256)	SMD: −0.329 (−0.540, −0.117)^∗^	*I*2 = 0%
	Adverse events (vs NSAIDs)	3 (623)	Or: 0.524 (0.121, 2.279)	*I*2 = 63.2%
Igho J. ONAKPOYA 2017 (UK) [32]	WOMAC scale (vs NSAIDs)	1 (331)	MD: −0.03 (−0.03, 0.09)	NA
Zhiqiang Wang 2021 (Australia) [33]	Pain (vs NSAIDs)	5 (648)	SMD = −0.09 (−0.30, 0.12)	*I*2 = 34.97%
	Function (vs NSAIDs)	3 (477)	SMD = −0.14 (−0.36, 0.09)	*I*2 = 20.02%
	Adverse events (vs NSAIDs)	3 (571)	RD: −0.12 (−0.24, −0.01)^∗^	*I*2 = 42.74%
	The use of rescue drugs (vs NSAIDs)	2 (443)	RD: 0.02 (−0.01, 0.04)	*I*2 = 0.01%
Jian Wu 2019 (China) [30]	WOMAC scale (vs NSAIDs)	1 (331)	SMD: −0.06 (−0.28, 0.15)	NA
	Adverse events (vs NSAIDs)	2 (159)	RR:0.81 (0.63, 1.05)	*I*2 = 0%
Liuting Zeng 2021 (China) [31]	VAS (vs NSAIDs)	2 (230)	MD: −0.34 (−1.25, 0.57)	*I*2 = 0%
	WOMAC (pain) scale (vs NSAIDs)	1 (331)	SMD: 0.04 (−0.18, 0.25)	NA
	WOMAC (physical) scale (vs NSAIDs)	1 (331)	SMD: 0.07 (−0.14, 0.29)	NA
	WOMAC (stiffness) scale (vs NSAIDs)	1 (331)	SMD: 0.07 (−0.17, 0.27)	NA
	Adverse events (vs NSAIDs)	3 (561)	RR: 0.55 (0.34, 0.88)^∗^	*I*2 = 70%
Weiyan Gong 2017 (China) [27]	VAS (vs NSAIDs)	1 (112)	MD: 13.00 (8.162,17.838)^∗^	NA
	WOMAC scale (vs NSAIDs)	1 (331)	MD: 0.13 (−0.302, 0.562)	NA
	Walking distance (vs NSAIDs)	2 (360)	MD: −1.17 (−19.7, 17.37)	*I*2 = 0%
	Adverse events (vs NSAIDs)	3 (491)	Or: 0.55 (0.38, 0.81)^∗^	*I*2 = 75.3%

*Note.* ^∗^The 95% confidence interval does not cross the invalid line.

**Table 3:** Result of the AMSTAR-2 assessments.

Author, year (country)	Q1	Q2	Q3	Q4	Q5	Q6	Q7	Q8	Q9	Q10	Q11	Q12	Q13	Q14	Q15	Q16	Quality
Raveendhara R. 2018 (USA) [26]	Y	Y	Y	Y	Y	Y	N	Y	Y	Y	Y	Y	Y	Y	N	Y	VL
James W. Daily 2016 (South Korea) [34]	Y	PY	Y	PY	Y	Y	N	Y	Y	Y	Y	Y	Y	N	Y	Y	VL
An-Fang Hsiao 2021 (China) [28]	Y	PY	Y	PY	Y	Y	N	Y	Y	Y	Y	Y	Y	Y	Y	Y	VL
Igho J. ONAKPOYA 2017 (UK) [32]	Y	PY	Y	Y	Y	Y	Y	Y	Y	Y	Y	Y	N	Y	N	Y	VL
Zhiqiang Wang 2021 (Australia) [33]	Y	PY	Y	Y	Y	Y	N	Y	Y	Y	Y	Y	Y	Y	Y	Y	VL
Wenli Dai 2021 (China) [29]	Y	Y	Y	Y	Y	Y	Y	Y	Y	Y	Y	Y	Y	Y	Y	Y	H
Jian Wu 2019 (China) [30]	Y	PY	Y	Y	Y	Y	N	Y	Y	Y	Y	Y	Y	Y	N	Y	VL
Liuting Zeng 2021 (China) [31]	Y	PY	Y	PY	Y	Y	N	Y	Y	Y	Y	Y	Y	Y	Y	Y	VL
Weiyan Gong 2017 (China) [27]	Y	PY	Y	Y	Y	Y	N	Y	Y	Y	Y	N	N	N	N	N	VL

*Note.* Y, yes; PY, partial yes; N, no; VL, very low; L, low; H, high.

**Table 4:** Results of the ROBIS assessments

Author, year (country)	Phase 1		Phase 2			Phase 3
	Assessing relevance	Domain 1: Study eligibility criteria	Domain 2: Identification and selection of studies	Domain 3: Collection and study appraisal	Domain 4: Synthesis and findings	Risk of bias in the review
Raveendhara R. 2018 (USA) [26]	√	√	√	√	×	√
James W. Daily 2016 (South Korea) [34]	√	√	×	√	×	√
An-Fang Hsiao 2021 (China) [28]	√	√	×	√	√	√
Igho J. ONAKPOYA 2017 (UK) [32]	√	√	√	√	×	×
Zhiqiang Wang 2021 (Australia) [33]	√	√	√	√	×	√
Wenli Dai 2021 (China) [29]	√	√	√	√	√	√
Jian Wu 2019 (China) [30]	√	√	√	√	×	√
Liuting Zeng 2021 (China) [31]	√	√	×	√	×	√
Weiyan Gong 2017 (China) [27]	√	√	√	×	×	×

Note:√, low risk; ×, high risk.

**Table 5:** Results of the PRISMA checklist.

Section/topic	Items	Raveendhara R. 2018 (USA) [26]	James W. Daily 2016 (South Korea) [34]	An-Fang Hsiao 2021 (China) [28]	Igho J. ONAKPOYA 2017 (UK) [32]	Zhiqiang Wang 2021 (Australia) [33]	Wenli Dai 2021 (China) [29]	Jian Wu 2019 (China) [30]	Liuting Zeng 2021 (China) [31]
Title	Q1. Title	Y	Y	Y	Y	Y	Y	Y	Y
Abstract	Q2. Structured summary	Y	Y	Y	Y	Y	Y	Y	Y
Introduction	Q3. Rationale	Y	Y	Y	Y	Y	Y	Y	Y
	Q4. Objectives	Y	Y	Y	Y	Y	Y	Y	Y
Methods	Q5. Protocol and registration	Y	N	N	N	N	Y	N	N
	Q6. Eligibility criteria	Y	Y	Y	Y	Y	Y	Y	Y
	Q7. Information sources	Y	Y	Y	Y	Y	Y	Y	Y
	Q8. Search	N	N	Y	Y	Y	N	N	Y
	Q9. Study selection	Y	Y	Y	Y	Y	Y	Y	Y
	Q10. Data collection process	Y	Y	Y	Y	Y	Y	Y	Y
	Q11. Data items	Y	Y	Y	Y	Y	Y	Y	Y
	Q12. Risk of bias in individual studies	Y	Y	Y	Y	Y	Y	Y	Y
	Q13. Summary measures	Y	Y	Y	Y	Y	Y	Y	Y
	Q14. Synthesis of results	Y	Y	Y	Y	Y	Y	Y	Y
	Q15. Risk of bias across studies	N	Y	Y	Y	Y	Y	Y	Y
	Q16. Additional analyses	Y	Y	Y	Y	Y	Y	Y	Y
Results	Q17. Study selection	Y	Y	Y	Y	Y	Y	Y	Y
	Q18. Study characteristics	Y	Y	Y	Y	Y	Y	Y	Y
	Q19. Risk of bias within studies	Y	Y	Y	Y	Y	Y	Y	Y
	Q20. Results of individual studies	Y	Y	Y	Y	Y	Y	Y	Y
	Q21. Synthesis of results	Y	Y	Y	Y	Y	Y	Y	Y
	Q22. Risk of bias across studies	N	Y	Y	N	Y	Y	Y	Y
	Q23. Additional analysis	Y	Y	Y	Y	Y	Y	Y	Y
Discussion	Q24. Summary of evidence	Y	Y	Y	Y	Y	Y	Y	Y
	Q25. Limitations	Y	Y	Y	Y	Y	Y	Y	Y
	Q26. Conclusions	Y	Y	Y	Y	Y	Y	Y	Y
Funding	Q27. Funding	Y	Y	Y	Y	Y	Y	Y	Y

*Note.* Y, yes; N, no.

**Table 6:** Results of evidence quality.

Author, year (country)	Outcomes	Studies (participants)	Limitations	Inconsistency	Indirectness	Imprecision	Publication bias	Quality
*A (CLE/C vs placebo)*								
Raveendhara R. 2018 (USA) [26]	Pain	5 (331)	0	0	0	0	−1④	Moderate
	Function	3 (232)	0	0	0	0	−1④	Moderate
	The use of rescue drugs	3 (141)	−1①	0	0	−1③	−1④	Very low
	Incidence of withdrawal from treatment due to adverse events	4 (288)	0	0	0	−1③	−1④	Low
	Adverse events	3 (247)	0	0	0	−1③	−1④	Low
James W. Daily 2016 (South Korea) [34]	VAS	3 (104)	0	0	0	−1③	0	Moderate
	WOMAC scale	3 (122)	0	−1②	0	−1③	0	Low
An-Fang Hsiao 2021 (China) [28]	VAS	7 (501)	0	−1②	0	−1③	−1④	Very low
	Adverse events	6 (527)	0	0②	0	−1③	0	Moderate
Igho J. ONAKPOYA 2017 (UK) [32]	VAS	5 (366)	−1①	−1②	0	0	−1④	Very low
	WOMAC scale	3 (167)	−1①	−1②	0	−1③	−1④	Very low
	LPFI	2 (107)	−1①	0	0	−1③	−1④	Very low
Zhiqiang Wang 2021 (Australia) [33]	Pain	12 (1,071)	0	−1②	0	0	−1④	Low
	Function	10 (973)	0	−1②	0	0	−1④	Low
	Adverse events	8 (791)	0	0	0	−1③	−1④	Low
	The use of rescue drugs	7 (300)	0	0	0	0	−1④	Moderate
	Analgesic discontinuation rate	4 (154)	0	−1②	0	−1③	−1④	Very low
Wenli Dai 2021 (China) [29]	VAS	8 (569)	0	−1②	0	0	0	Moderate
	WOMAC scale	5 (377)	0	−1②	0	0	0	Moderate
	WOMAC (pain) scale	5 (377)	0	−1②	0	0	0	Moderate
	WOMAC (physical) scale	5 (377)	0	−1②	0	0	0	Moderate
	WOMAC (stiffness) scale	5 (377)	0	−1②	0	0	0	Moderate
	Adverse events	7 (623)	0	0	0	−1③	0	Moderate
Jian Wu 2019 (China) [30]	WOMAC scale	3 (146)	0	0	0	−1③	−1④	Low
	VAS	2 (98)	0	0	0	−1③	−1④	Low
	Adverse events	2 (113)	0	0	0	−1③	−1④	Low
Liuting Zeng 2021 (China) [31]	VAS	6 (381)	−1①	0	0	0	0	Moderate
	WOMAC (pain) scale	4 (315)	−1①	0	0	0	−1⑤	Low
	WOMAC (physical) scale	4 (315)	−1①	−1②	0	0	0	Low
	WOMAC (stiffness) scale	4 (315)	−1①	0	0	0	0	Moderate
	Adverse events	6 (629)	−1①	0	0	−1③	0	Low
Weiyan Gong 2017 (China) [27]	VAS	2 (82)	−1①	0	0	−1③	−1④	Very low
	WOMAC scale	2 (82)	−1①	0	0	−1③	−1④	Very low
	Adverse events	2 (152)	−1①	0	0	−1③	−1④	Very low
	Walking distance	1 (48)	−1①	−1②	0	−1③	−1④	Very low
*B (CLE/C vs CT)*								
Raveendhara R. 2018 (USA) [26]	Pain (vs NSAIDs)	2 (422)	0	0	0	−1③	−1④	Low
	Function (vs NSAIDs)	1 (331)	0	−1②	0	−1③	−1④	Very low
	The use of rescue drugs (vs NSAIDs)	2 (422)	0	0	0	−1③	−1④	Low
	Incidence of withdrawal from treatment due to adverse events (vs NSAIDs)	2 (474)	0	0	0	0	−1④	Moderate
	Adverse events (vs NSAIDs)	2 (467)	0	0	0	0	−1④	Moderate
James W. Daily 2016 (South Korea) [34]	WOMAC scale (vs painkillers)	5 (625)	0	−1②	0	−1③	0	Low
An-Fang Hsiao 2021 (China) [28]	VAS (vs NSAIDs)	2 (256)	0	0	0	0	−1⑤	Moderate
	Adverse events (vs NSAIDs)	3 (623)	0	0	0	−1③	0	Moderate
Igho J. ONAKPOYA 2017 (UK) [32]	WOMAC scale (vs NSAIDs)	1 (331)	0	−1②	0	−1③	−1④	Very Low
Zhiqiang Wang 2021 (Australia) [33]	Pain (vs NSAIDs)	5 (648)	0	0	0	−1③	−1④	Low
	Function vs NSAIDs)	3 (477)	0	0	0	−1③	−1④	Low
	Adverse events (vs NSAIDs)	3 (571)	0	0	0	0	−1④	Moderate
	The use of rescue drugs (vs NSAIDs)	2 (443)	0	0	0	−1③	−1④	Low
Jian Wu 2019 (China) [30]	WOMAC scale (vs NSAIDs)	1 (331)	0	−1②	0	−1③	−1④	Very low
	Adverse events (vs NSAIDs)	2 (159)	0	0	0	−1③	−1④	Low
Liuting Zeng 2021 (China) [31]	VAS (vs NSAIDs)	2 (230)	−1①	0	0	−1③	0	Low
	WOMAC (pain) scale (vs NSAIDs)	1 (331)	−1①	−1②	0	−1③	−1④	Very low
	WOMAC (physical) scale (vs NSAIDs)	1 (331)	−1①	−1②	0	−1③	−1④	Very low
	WOMAC (stiffness) scale (vs NSAIDs)	1 (331)	−1①	0	0	−1③	−1④	Very low
	Adverse events (vs NSAIDs)	3 (561)	−1①	0	0	0	0	Moderate
Weiyan Gong 2017 (China) [27]	VAS (vs NSAIDs)	1 (112)	−1①	−1②	0	−1③	−1④	Very low
	WOMAC scale (vs NSAIDs)	1 (331)	−1①	−1②	0	−1③	−1④	Very low
	Walking distance (vs NSAIDs)	2 (360)	−1①	0	0	−1③	−1④	Very low
	Adverse events (vs NSAIDs)	3 (491)	−1①	−1②	0	0	−1④	Very low

*Note.* ①The included studies have a large bias in methodology such as randomization, allocation concealment, and blinding. ②The confidence interval overlaps less, or the I2 value of the combined results was larger. ③The sample size from the included studies does not meet the optimal sample size or the 95% confidence interval crosses the invalid line. ④The funnel chart is asymmetry. ⑤Fewer studies were included, and their results were all positive, which may result in a large publication bias.

## Data Availability

The datasets analyzed during the current study are available from the corresponding author on reasonable request.

## Supplementary Materials

Supplementary Materials The SR/MA overview is based on the guidelines specified in Cochrane Handbook [19], the Preferred Reporting Project for System Reviews and Meta-Analyses (PRISMA) statement (Supplementary file 1) [20], and the overview of high-quality methods [21, 22]. Supplementary file 2 provided the search strategy.
