# Supplementary material for: Quality of Evidence Supporting the Role of Curcuma Longa Extract/Curcumin for the Treatment of Osteoarthritis: An Overview of Systematic Reviews
Source: Evid Based Complement Alternat Med. 2022 Mar 31;2022:6159874. doi: 10.1155/2022/6159874 (PMC8991401; doi:10.1155/2022/6159874)
Supplement: Supplementary Materials — The SR/MA overview is based on the guidelines specified in Cochrane Handbook [19], the Preferred Reporting Project for System Reviews and Meta-Analyses (PRISMA) statement (Supplementary file 1) [20], and the overview of high-quality methods [21, 22]. Supplementary file 2 provided the search strategy. [file 6159874.f1.zip › 6159874.f1/Supplementary file 2 (1).docx]

| **Query** | **Search term** |
| --- | --- |
| #1 | “Curcumin” OR “Curcumas” OR “Tumeric” OR “Tumerics” OR “Turmeric” OR “Turmerics” OR “Curcuma zedoaria” OR “Curcuma zedoarias” OR “zedoaria, Curcuma” OR “Zedoary zedoaria” OR “Zedoary zedoarias” OR “zedoaria, Zedoary” OR “Curcuma longa” OR “Curcuma longas” OR “longa, Curcuma” OR “Curcuma Longa” OR “Turmeric Yellow” OR “Yellow, Turmeric” OR “Diferuloylmethane” |
| #2 | “Osteoarthritis”[Mesh] |
| #3 | “Osteoarthritis” OR “Osteoarthritides” OR “Osteoarthrosis” OR “Osteoarthroses” OR “Arthritis, Degenerative” OR “Arthritides, Degenerative” OR “Degenerative Arthritides” OR “Degenerative Arthritis” OR “Arthrosis” OR “Arthroses” OR "Osteoarthrosis Deformans” |
| #4 | #2 OR #3 |
| #5 | Meta-Analysis as Topic [Mesh] |
| #6 | “Systematic review” OR “meta-analysis” OR “meta analysis” OR “meta-analyses” OR "Review, Systematic” |
| #7 | #5 OR #6 |
| #8 | #1 AND #4 AND #7 |

Search strategy for the PubMed database.

| **Query** | **Search term** |
| --- | --- |
| #1 | Curcumin OR "Curcumas" OR "Tumeric" OR "Tumerics" OR "Turmeric" OR "Turmerics" OR "Curcuma zedoaria" OR "Curcuma zedoarias" OR "zedoaria, Curcuma" OR "Zedoary zedoaria" OR "Zedoary zedoarias" OR "zedoaria, Zedoary" OR "Curcuma longa" OR "Curcuma longas" OR "longa, Curcuma" OR "Curcuma Longa" OR "Turmeric Yellow" OR "Yellow, Turmeric" OR "Diferuloylmethane" |
| #2 | [Osteoarthritis] explode all trees |
| #3 | Arthrosis OR "Arthroses" OR "Arthritides, Degenerative" OR "Osteoarthroses" OR "Degenerative Arthritis" OR "Osteoarthritides" OR "Osteoarthrosis" OR "Degenerative Arthritides" OR "Arthritis, Degenerative" OR "Osteoarthrosis Deformans" OR "Osteoarthritis" |
| #4 | #2 OR #3 |
| #5 | "Systematic review" OR "meta-analysis" OR "meta analysis" OR "meta-analyses" OR "Systematic reviews" OR "meta" OR ""meta analyses" |
| #6 | #1 AND #4 AND #5 |

Search strategy for the Cochrane Library database.

| **Query** | **Search term** |
| --- | --- |
| #1 | Curcumin OR "Curcumas" OR "Tumeric" OR "Tumerics" OR "Turmeric" OR "Turmerics" OR "Curcuma zedoaria" OR "Curcuma zedoarias" OR "zedoaria, Curcuma" OR "Zedoary zedoaria" OR "Zedoary zedoarias" OR "zedoaria, Zedoary" OR "Curcuma longa" OR "Curcuma longas" OR "longa, Curcuma" OR "Curcuma Longa" OR "Turmeric Yellow" OR "Yellow, Turmeric" OR "Diferuloylmethane" |
| #2 | osteoarthritis'/exp |
| #3 | Arthrosis OR "Arthroses" OR "Arthritides, Degenerative" OR "Osteoarthroses" OR "Degenerative Arthritis" OR "Osteoarthritides" OR "Osteoarthrosis" OR "Degenerative Arthritides" OR "Arthritis, Degenerative" OR "Osteoarthrosis Deformans" OR "Osteoarthritis" |
| #4 | #2 OR #3 |
| #5 | "Systematic review" OR "meta-analysis" OR "meta analysis" OR "meta-analyses" OR "Systematic reviews" OR "meta" OR ""meta analyses" |
| #6 | #1 AND #4 AND #5 |

Search strategy for the Embase database.

SU=(姜黄素+姜黄提取物+姜黄) AND SU=(骨关节炎+关节炎+骨性关节炎+痹症+骨痹) AND SU=(系统评价+meta分析+meta+荟萃分析)

Search strategy for the CNKI database.

主题:（姜黄素 or 姜黄提取物 or 姜黄）*主题:(骨关节炎 or 关节炎 or 骨性关节炎 or 痹症 or 骨痹) *主题:(系统评价 or meta分析 or meta or 荟萃分析)

Search strategy for the Wanfang database.

(M=姜黄素+M=姜黄提取物+M=姜黄)*(M=骨关节炎+M=关节炎+M=骨性关节炎+M=痹症+M=骨痹)*(M=系统评价+M=meta分析+M=meta+M=荟萃分析)

Search strategy for the Chongqing VIP database.

(姜黄素 OR 姜黄提取物 OR 姜黄) AND (骨关节炎 OR 关节炎 OR 骨性关节炎 OR 痹症 OR 骨痹) AND (系统评价 OR meta分析 OR meta OR 荟萃分析)

Search strategy for the Chongqing Chinese Biological Medicine (CBM) database.
